# Biosynthetic pathway deflection – a new cell line engineering approach

**DOI:** 10.1186/1753-6561-5-S8-O4

**Published:** 2011-11-22

**Authors:** Hans Henning von Horsten, Thomas Rose, Volker Sandig

**Affiliations:** 1 ProBioGen AG, Berlin, Germany

## 

With increasing information on genome, transcriptome and metabolome of commonly used production cell lines, engineering becomes an increasingly popular approach to achieve desired product attributes, growth behavior and nutrient consumption. Tools range from feeding intermediate metabolites, overexpression or deregulation of key enzymes of a pathway to knock-out and RNA silencing. While conceptionally simple, the latter approaches are either labor intensive or costly to apply at large scale.

## Fucose targeted glycoengineering

**Figure 1:**
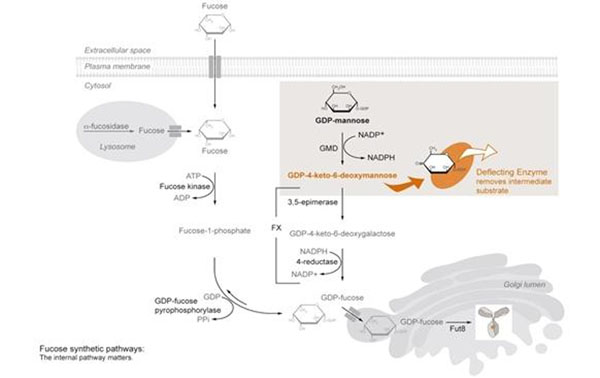
Overview of GDP-L-Fucose Biosynthesis showing the point of substrate deflection within the fucose de-novo synthesis pathway.

Aiming at glycan modulation we added another principle to this toolbox: enzymatic deflection of a biochemical pathway. Fucose is synthesized inside the cell from GDP-mannose via short lived intermediates before it is transported to the Golgi apparatus for attachment to the nascent glycan (Figure 1). A bacterial enzyme is used to redirect synthesis towards a heterologous activated hexose that cannot be utilized by the cell resulting in depletion of the natural pathway (deflecting enzyme, Figure 1). To our surprise, even lowest level expression of the enzyme completely abolishes fucose synthesis in stably modified cells.

The approach allows producing antibodies that are devoid of core fucose at Fc glycans of the CH2 domain [1]. This modification provides higher flexibility to the Fc-region of IgG1 antibodies and enhances their binding to the FcγRIIIa receptor of NK cells - the dominating effector cells in antibody dependent cytotoxicity (ADCC). Consequently, the potency of antibodies directed against tumor or infected cells is substantially increased.

In contrast to other strategies the approach is easily applied to the starter cell line of choice and, moreover, allows modification of fully developed producer cell lines within weeks.

## Simultaneous regulation of multiple cellular pathways

Another concept for clone engineering is based on simultaneous modulation of multiple cellular processes. We found that the Rho GTPase cdc42 is a highly suitable effector molecule for this purpose. This pleiotropic modulator dramatically boosts antibody titers when overexpressed in the cytosol of pharmaceutical producer clones (Table1).

**Table 1:** CDC42-mediated relative mAb-titer increase over native clone titers. The clones represent five different products.

Titers of Naïve mAb producing clones [g/l]	Titers of cdc42-engineered mAb producing clones [g/l]	Relative Fold Increase per modified clone
0,8	1,65	**2,06**
0,9	2,2	**2,4**
2,3	3,0	**1,3**
2,6	4,5	**1,73**
0,8	1,65	**2,06**
